# Supplementary material for: A Lotus japonicus E3 ligase interacts with the Nod Factor Receptor 5 and positively regulates nodulation
Source: BMC Plant Biol. 2018 Oct 3;18:217. doi: 10.1186/s12870-018-1425-z (PMC6171183; doi:10.1186/s12870-018-1425-z)
Supplement: Supplementary file 1 — Table S1. Primers used for cloning into expression vectors. Table S2. Primers used in qRT-PCR. Table S3. Primers used for cloning into GoldenGate and Gateway vectors. Figure S1. Phylogenetic tree of amino acid sequences of LjPUB13 with previously characterized E3s of other species and L. japonicus uncharacterized PUBs. Figure S2. Amino acid sequence alignment of LjPUB13 with AtPUB13. Figure S3. Expression levels of LjPUB13 in pub13 LORE1 mutants. Figure S4. Formation of nodules in pub13.1 vs. har1 mutants. Figure S5. Inoculated 28-day-old wild type and homozygous pub13.3 mutants. Figure S6. Nodule sections of wild type, pub13.1 and pub13.2 plants. Figure S7. ROS accumulation in roots of pub13 mutants vs wt plants. Figure S8. Expression of defence genes in L. japonicus wild type and pub13 mutants. Figure S9. LjFLS2 ubiquitination tests. (DOCX 39900 kb) [file 12870_2018_1425_MOESM1_ESM.docx]

**SUPPLEMENTAL INFORMATION**

**Table S1**: Primers used for cloning into protein expression vectors

|  | Forward | Reverse |
| --- | --- | --- |
| **Cloning in pGEX-6P-1** | | |
| *Lj*PUB13 | 5’-ACGGAATTCATGGAGGTTGTTGTTGAG-3’ | 5’-AGCTCTCGAGTCAGGTATCATCAAGGTTAG-3’ |
| *Lj*BAK1_cyt_ | 5’-ACGGAATTCTATTTGCGAAAAAAGAAACC-3’ | 5’-AGCTCTCGAGTCATCTAGGGCCTGATAG-3’ |
| *Lj*FLS2_cyt_ | 5’-ACAGAATTCCGACGCACCAGACTCAGA-3’ | 5’-ACGTCTCGAGCTATTTTTCTGTTTGAAGC-3’ |
| **Cloning in pET-21a** | | |
| *Lj*PUB13 | 5’-ACGGAATTCATGGAGGTTGTTGTTGAG-3’ | 5’-AGCTCTCGAGGGTATCATCAAGATTAGA-3’ |

**Table S2**: Primers used in qRT-PCR

|  | Forward | Reverse |
| --- | --- | --- |
| ***LjPUB13*** | 5’-ATGAAACTGCTGACAGAACCAA-3’ | 5’-TCACCAGAAGAAAGGTGCACTA-3’ |
| ***LjBAK1*** | 5’-TACCTGTCAACTGGAAAGTCTTCCG-3’ | 5’-GTCTTTCAGAAGTCCTTTAACCCAA-3’ |
| ***LjFLS2*** | 5’-CACAGTTGGTTATTTGGCACCAGAA-3’ | 5’-TGCAAGGGCTCTTGCGACTACTTCA-3’ |
| ***LjMPK3*** | 5’-ATTGATCCCACCAAAAGAATCACAGTTGAA-3’ | 5’-CCAATGCTTCCCTGTAGATCATCTC-3’ |
| ***LjPEROXIDASE*** | 5’-TGTGCGGAGGTACTTGGGCTTAAGA-3’ | 5’-CTTCCCAGGCAAATGCCAACACTAG-3’ |
| ***LjPR1a*** | 5’-AAGGTCAGGTGCGACAATAATCGCG-3’ | 5’-TACAAACGTGCCTACCAAGAAACTG-3’ |
| ***LjATP*** | 5’-CAATGTCGCCAAGGCCCATGGTG-3’ | 5’-AACACCACTCTCGATCATTTCTCTG |
| ***LjUBQ*** | 5’-ATGTGCATTTTAAGACAGGG-3’ | 5’-GAACGTAGAAGATTGCCTGAA-3’ |
| ***LjPP2*** | 5’-GTAAATGCGTCTAAAGATAGGGTCC-3’ | 5’-ACTAGACTGTAGTGCTTGAGAGGC-3’ |

**Table S3:** Primers used for cloning into GoldenGate (GG) and Gateway (GW) vectors

| ***LjPUB13* promoter** (GG) | |
| --- | --- |
| forward | 5’-TTTGAAGACAAGGAGGTCGATTCATGTTTTGCACC-3’ |
| reverse | 5’-TTTGAAGACAACATTGGCTGCGCCGCCACCACCAC-3’ |
| ***LjPUB13* terminator** (GG) | |
| forward | 5’-TTTGAAGACAAGCTTGATGTCTTGTTTATACTTTTG-3’ |
| reverse | 5’-TTTGAAGACAAAGCGCCAACATATTAGATAAGTTC-3’ |
| ***PUB13_ARM_* in pGREEN029:35S:GW:nYFP/cYFP** (GW) | |
| forward | 5’-GGGGACAAGTTTGTACAAAAAAGCAGGCTTCATGAGCACCTCTCAACCTAGC-3’ |
| reverse | 5’-GGGGACCACTTTGTACAAGAAAGCTGGGTCGGTATCATCAAGGTTAGAAATTG-3’ |
| ***LjBAK1* in pGREEN029:35S:GW:nYFP/cYFP** (GW) | |
| forward | 5’-CACCATGGAGAGAGTGAATTCATC-3’ |
| reverse | 5’-TCTAGGGCCTGATAGTTCATCTGGC-3’ |





**Figure S1.** Neighbor-joining phylogenetic tree of amino acid sequences of *Lj*PUB13 (Lj3g3v3189730.1) with *Lotus japonicus* uncharacterized proteins detected in *Lotus* base v.3.0 showing the strongest similarity with *Lj*PUB13, and previously characterized E3s of other species: *At*PUB4 (NP_179895), *At*PUB12 (NP_565676), *At*PUB13 (NP_190235), *At*PUB14 (NP_191045), *At*PUB15 (NP_199049), *At*PUB17 (NP_174228), *At*PUB18 (NP_172526), *At*PUB19 (NP_176225), *At*PUB22 (NP_190813), *At*PUB23 (NP_181137), *At*PUB24 (NP_566402), *At*SAUL (NP_564125), *Bn*ARC1 (AGP76183), *Mt*PUB1 (DDA33939), *Mt*LIN (XP_003591660), *Nt*PUB4 (XP_009797979), *Os*SPL11 (Q0IMG9), *Lj*CERBERUS (C6L7U1), *Lj*SINA4 (CCG06554). *Lj*PUB13 is the closest homologue to *Arabidopsis* PUB13. The alignment and phylogenetic analysis of the deduced amino acid sequences were performed with CLC sequence viewer, v.6.7.1 (CLC bio A/S). The multiple alignment parameters were adjusted with gap cost 10 and gap extension 1. The phylogenetic trees were constructed using the neighbor-joining algorithm with bootstrap analysis of 1000 replicates.





**Figure S2.** Amino acid sequence alignment of *L. japonicus* (*Lj*) PUB13 with *Arabidopsis* (*At*) PUB13. Conserved U-box and ARM repeat regions are marked as boxes and black lines, respectively. The *Lj*PUB13_ARM_ domain used in the split-YFP experiments is also marked. Protein sequences were aligned using clustalW2 (EMBL-EBI). Protein domains were identified with ScanProsite (SIB Swiss Institute of Bioinformatics).


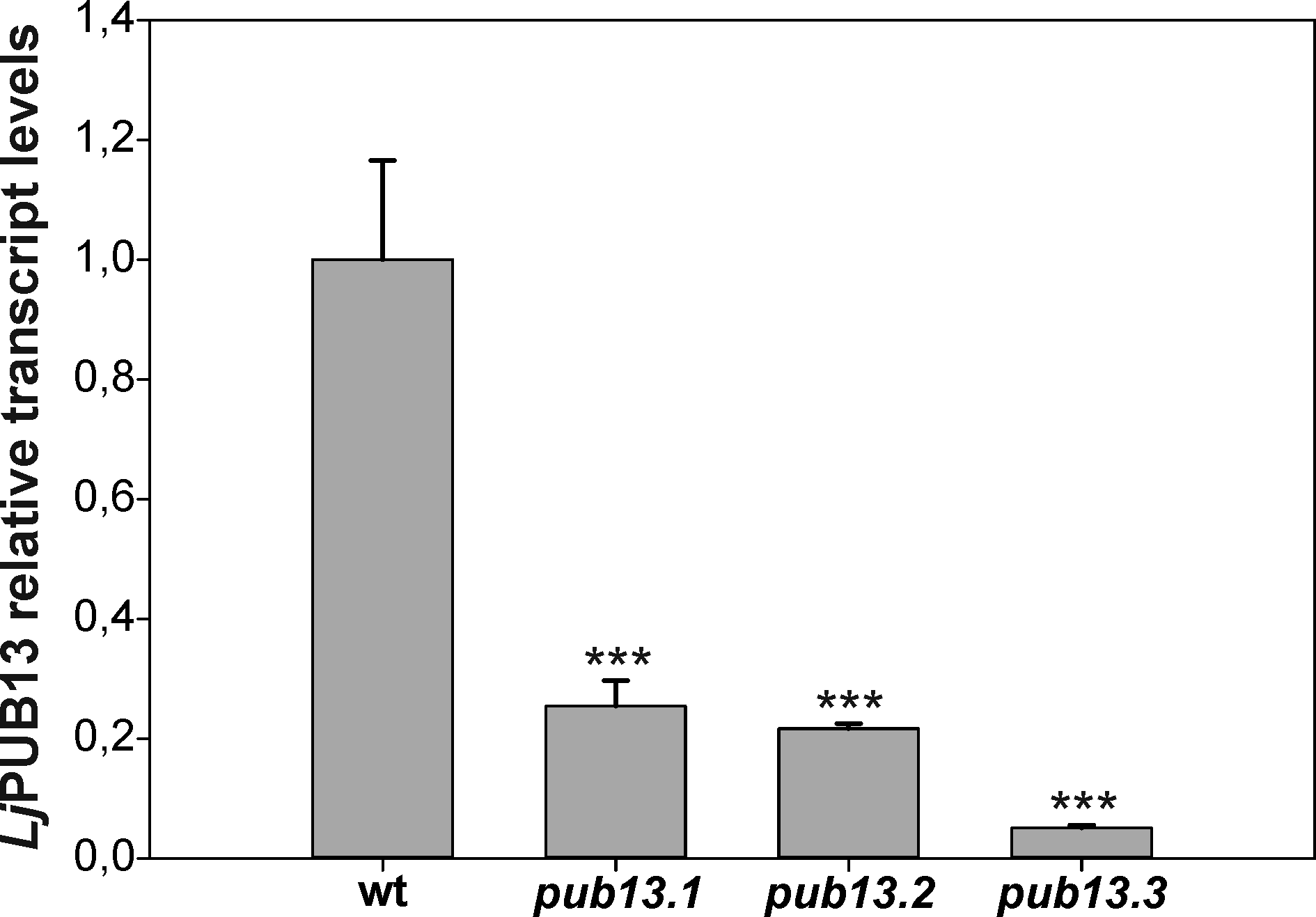


**Figure S3.** Expression levels of *Lj*PUB13 in *pub13* LORE1 mutants. Transcript levels were normalized to ubiquitin (*UBQ*). Bars represent means (+SE) of three biological replications (n=10). Comparisons are between each mutant with the wild type (wt) (P<0.001).


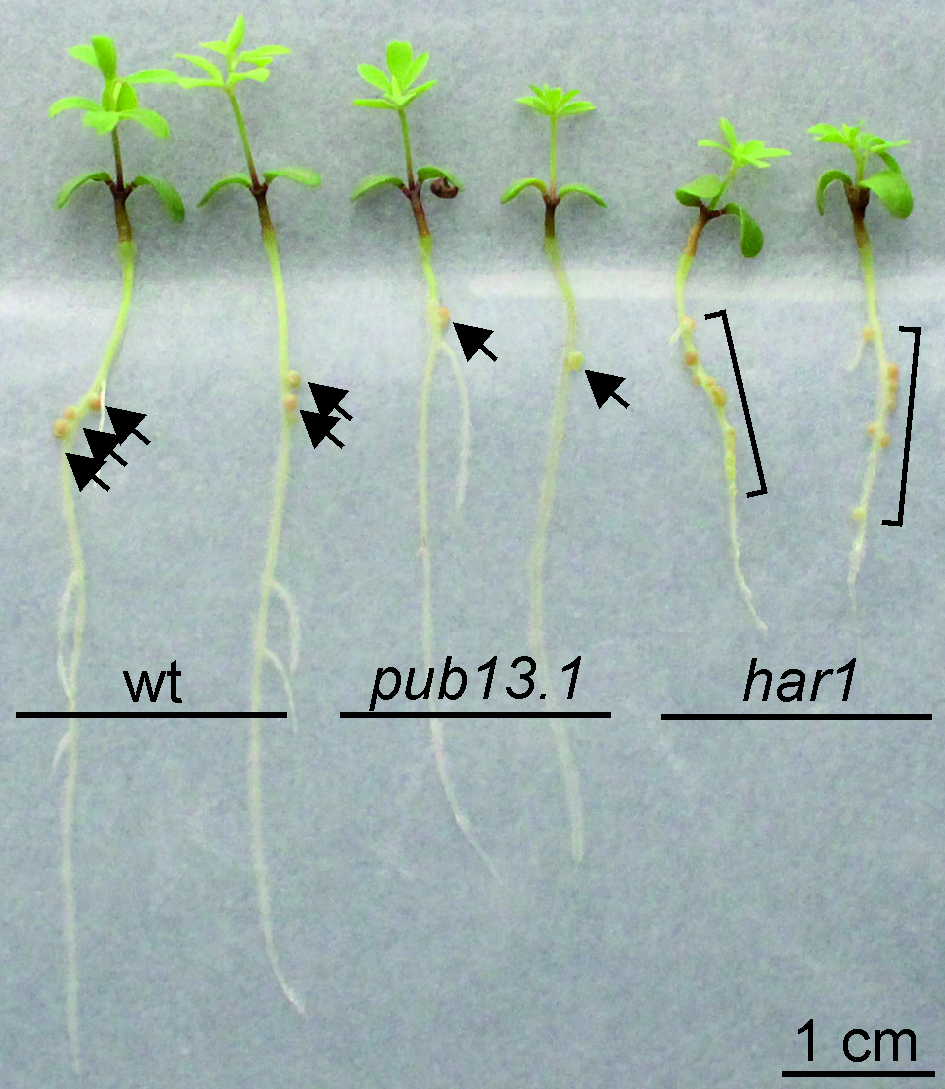


**Figure S4.** Formation of nodules in *pub13.1* *vs. har1* mutants. Arrows show nodules in wild-type and *pub13.1* mutant and brackets show the extended nodulation zone with many nodules in the *har1* mutant.


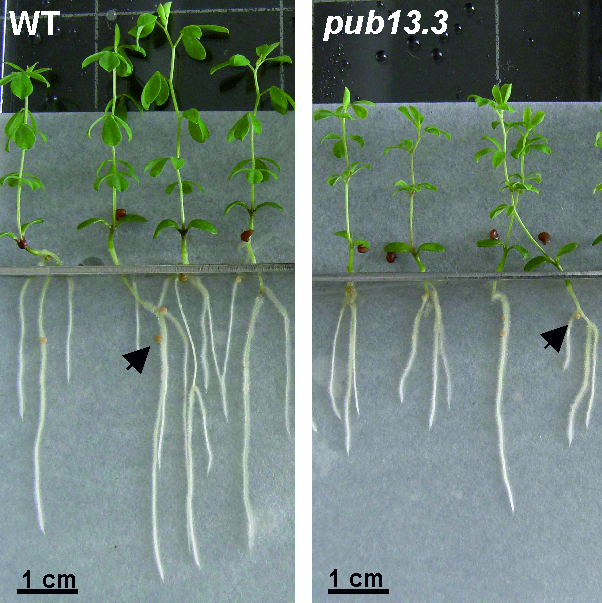


**Figure S5.** Inoculated 28-day-old wt and homozygous *pub13.3* mutants grown under normal conditions. Arrows show nodules.


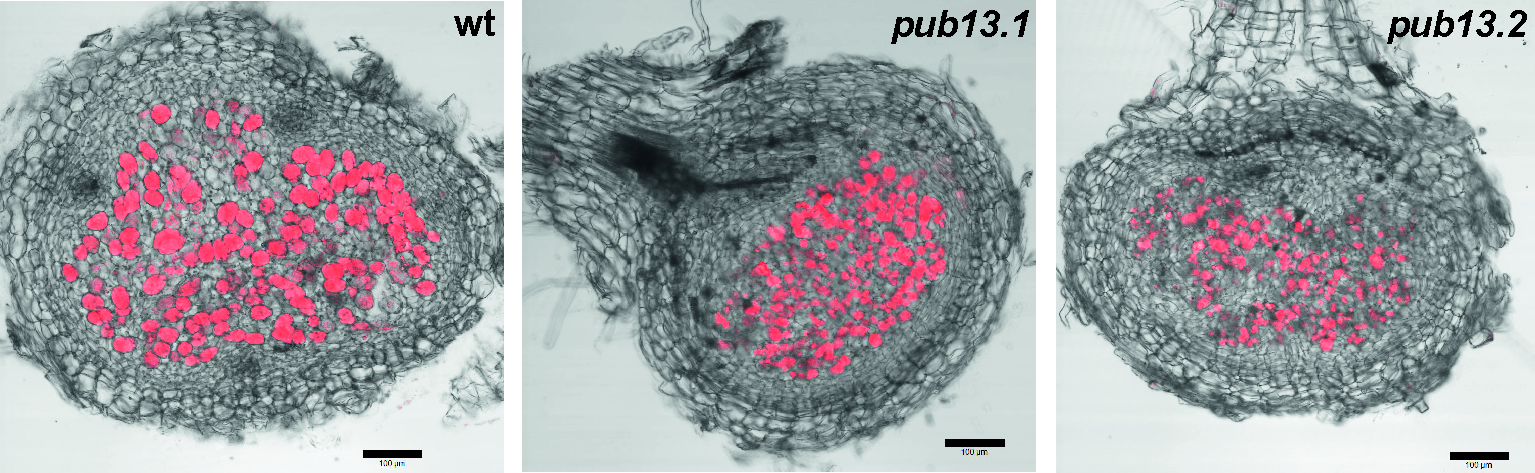


**Figure S6.** Nodule sections of wt, *pub13.1* and *pub13.2* plants, 28 days post inoculation by the *M. loti* strain R7A expressing the DsRed gene.





**Figure S7.** ROS accumulation in roots of *pub13* mutants *vs* wt plants. ROS burst in wt, *pub13.1* and *pub13.2* roots triggered by flg22. *Lotus* roots were treated with either water (mock) or 0.5 μM flg22. The data are shown as means from 15 roots. The differences between wt and *pub13* mutants are not statistically significant (*P*>0.05).


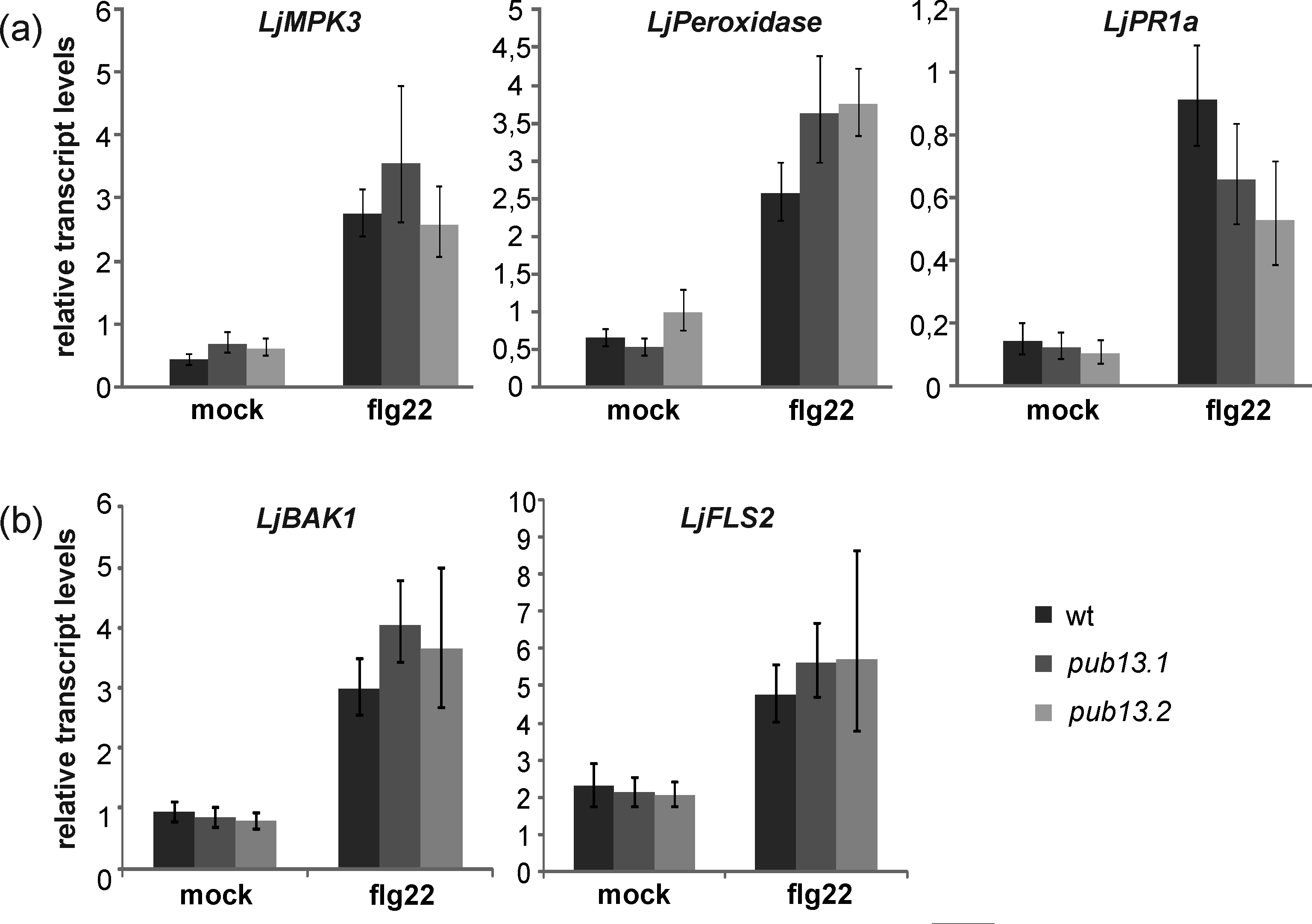


**Figure S8.** Expression levels of immune responsive genes (a), *LjFLS2* and *LjBAK1* genes (b), in *L. japonicus* wt and *pub13* mutants. Ten-day-old plants were treated with either water (mock) or 1 μM flg22 for 1 hour. Transcript levels were normalized to ubiquitin (*UBQ*), protein phosphatase 2A (*PP2*) and ATP-synthase (*ATP*) reference genes. Bars represent means (+SE) of three biological replications (n=10).


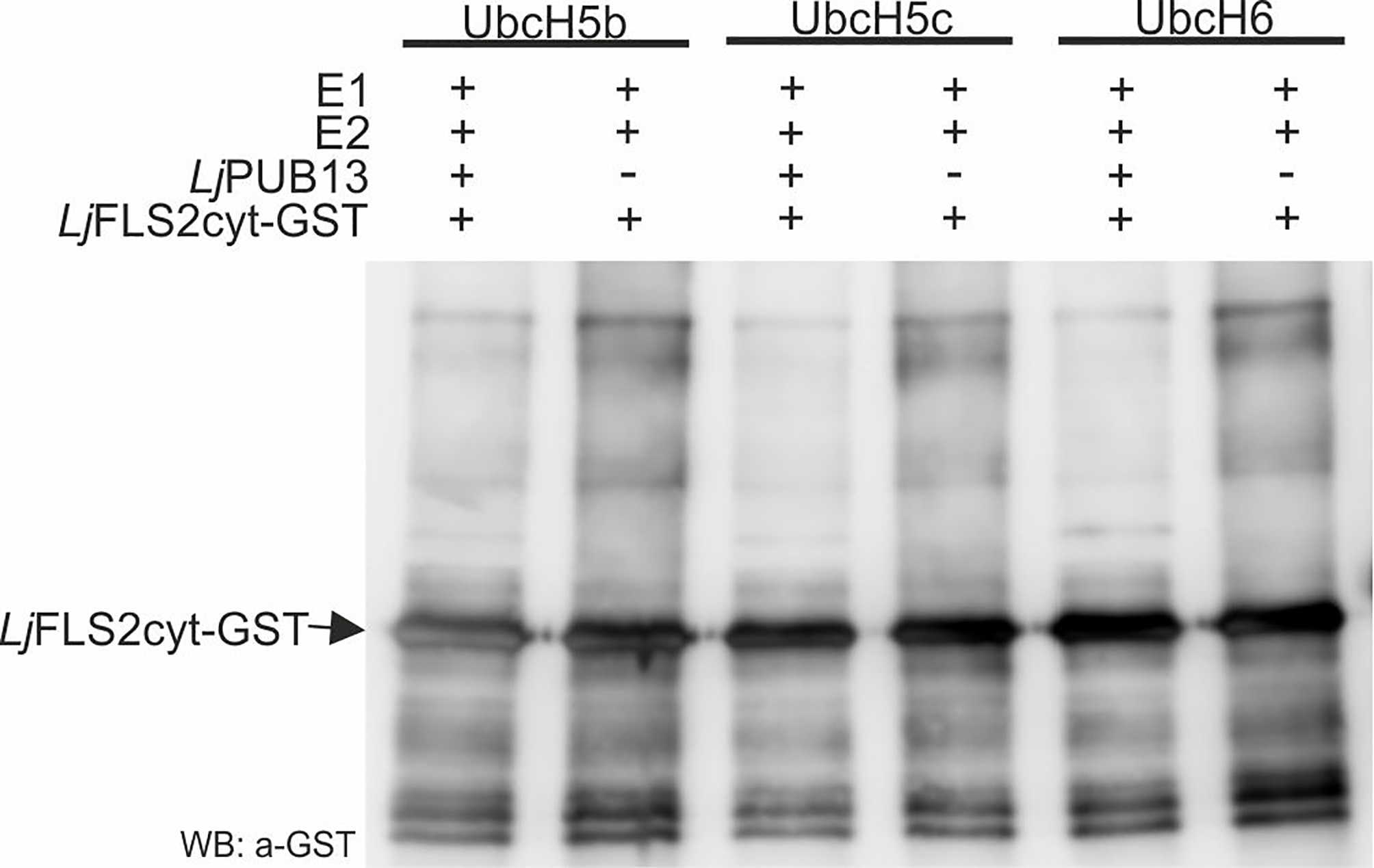


**Figure S9.** *Lj*FLS2 ubiquitination tests. *Lj*FLS2 was purified as a GST fusion protein, while an untagged version of *Lj*PUB13 was used. Three E2 enzymes were tested: UbcH5b, UbcH5c and UbcH6. An anti-GST antibody was used to detect ubiquitination. *Lj*FLS2 ubiquitination was not observed.
